# Synthetic Routes and Clinical Application of Representative Small-Molecule EGFR Inhibitors for Cancer Therapy

**DOI:** 10.3390/molecules29071448

**Published:** 2024-03-23

**Authors:** Ya-Tao Wang, Peng-Cheng Yang, Jing-Yi Zhang, Jin-Feng Sun

**Affiliations:** 1 First People’s Hospital of Shangqiu, Shangqiu 476100, China; 2 Key Laboratory of Natural Medicines of the Changbai Mountain, Ministry of Education, College of Pharmacy, Yanbian University, Yanji 133002, China; 0000008827@ybu.edu.cn; 3 College of Chemistry and Chemical Engineering, Zhengzhou Normal University, Zhengzhou 450044, China; zhangjingyi@zznu.edu.cn

**Keywords:** EGFR, tyrosine kinase inhibitors (TKIs), small molecule, synthetic routes, application

## Abstract

The epidermal growth factor receptor (EGFR) plays a pivotal role in cancer therapeutics, with small-molecule EGFR inhibitors emerging as significant agents in combating this disease. This review explores the synthesis and clinical utilization of EGFR inhibitors, starting with the indispensable role of EGFR in oncogenesis and emphasizing the intricate molecular aspects of the EGFR-signaling pathway. It subsequently provides information on the structural characteristics of representative small-molecule EGFR inhibitors in the clinic. The synthetic methods and associated challenges pertaining to these compounds are thoroughly examined, along with innovative strategies to overcome these obstacles. Furthermore, the review discusses the clinical applications of FDA-approved EGFR inhibitors such as erlotinib, gefitinib, afatinib, and osimertinib across various cancer types and their corresponding clinical outcomes. Additionally, it addresses the emergence of resistance mechanisms and potential counterstrategies. Taken together, this review aims to provide valuable insights for researchers, clinicians, and pharmaceutical scientists interested in comprehending the current landscape of small-molecule EGFR inhibitors.

## 1. Introduction

EGFR, also known as ErbB1 or HER1, belongs to the ErbB family of receptor tyrosine kinases (RTKs). Structurally, EGFR consists of an extracellular ligand-binding domain, a single transmembrane domain, an intracellular tyrosine kinase domain, and a C-terminal tail [1]. Upon ligand binding, EGFR undergoes conformational changes that lead to dimerization and activation of its intrinsic kinase activity, resulting in autophosphorylation of specific tyrosine residues within the receptor’s intracellular domain. This phosphorylation event initiates downstream signaling cascades involved in cell proliferation, survival, and differentiation. Crystallographic studies have provided detailed insights into the structure of EGFR and its interactions with ligands and inhibitors [2]. The crystal structure of the EGFR kinase domain complexed with inhibitors such as gefitinib and erlotinib has been elucidated, revealing the binding mode of these small molecules within the ATP-binding pocket of the kinase domain [3,4]. Additionally, structural studies have elucidated the mechanisms of resistance mutations, such as the T790M gatekeeper mutation, which affects the binding affinity of certain EGFR inhibitors [5,6,7]. The identification and advancement of small-molecule inhibitors aimed at the EGFR have significantly transformed the therapeutic paradigm across multiple cancer types, notably within non-small-cell lung cancer (NSCLC) [8,9,10]. EGFR inhibitors have demonstrated remarkable clinical efficacy, significantly improving patient outcomes. This review delves into the synthesis and application of representative small-molecule EGFR inhibitors, shedding light on the historical evolution, mechanisms of action, pharmacological properties, and therapeutic implications of these agents.

The journey of EGFR inhibitors began with the identification of EGFR as a key player in cancer progression. Subsequent research efforts led to the discovery of small molecules capable of inhibiting EGFR signaling. Significant strides in the advancement of EGFR inhibitors encompass pivotal landmarks marked by the discovery of activating mutations, notably the L858R substitution and Exon 19 deletions, which render tumors highly responsive to these agents [11]. The advent of initial EGFR inhibitors such as gefitinib and erlotinib represented a pivotal advancement, presenting a promising prospect for individuals afflicted with EGFR-mutated NSCLC [12]. EGFR inhibitors exert their primary mechanism through the inhibition of the tyrosine kinase activity inherent in EGFR. This action effectively hampers the progression of downstream signaling cascades crucially implicated in processes related to cell proliferation, survival, and the regulation of angiogenesis [13]. The interaction between these diminutive compounds and the intracellular kinase domain of EGFR obstructs the process of autophosphorylation, consequently impeding the activation of subsequent cascades, including the Raf/Ras/ERK/MEK and PI3K/Akt/mTOR pathways [14]. Additionally, third-generation EGFR inhibitors like osimertinib exhibit unique mechanisms of action, effectively targeting T790M resistance mutations in EGFR [15].

Understanding the pharmacological properties of EGFR inhibitors is crucial for optimizing their clinical use. Factors like pharmacokinetics, bioavailability, and drug–drug interactions influence dosing regimens and treatment outcomes. Some EGFR inhibitors, like afatinib, are irreversible binders, while others, like osimertinib, are additionally highly selective for mutant EGFR variants, minimizing off-target effects [16]. These agents exhibit varying pharmacokinetic profiles, which impact their efficacy and tolerability [17].

**Figure 1:**
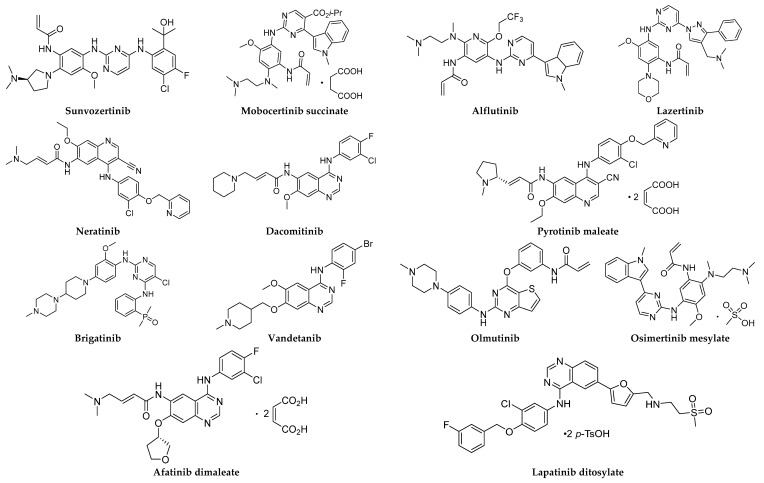

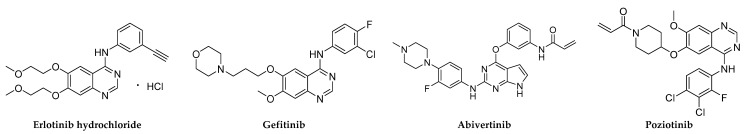
Chemical structures of small-molecule EGFR inhibitors.

The advent of clinically endorsed EGFR inhibitors has revolutionized the landscape of cancer treatment, yielding significant enhancements in both progression-free survival and overall survival among individuals afflicted with EGFR-mutated tumors [18]. These therapies have become standard-of-care for EGFR-positive NSCLC and are also being explored in other malignancies with EGFR alterations. Moreover, recent advances in liquid biopsies and patient stratification enable precise identification of EGFR mutations, facilitating personalized treatment approaches [19]. The synthesis and application of representative small-molecule EGFR inhibitors are regarded as a remarkable achievement in the field of oncology (Table 1 and Figure 1). These agents have redefined cancer treatment paradigms and significantly improved patient outcomes. Understanding their historical evolution, mechanisms of action, pharmacological properties, and therapeutic implications is essential for optimizing their use and driving further advancements in precision medicine.

Among the current tyrosine kinase inhibitors (TKIs) targeting EGFR, the majority were developed with the aid of structure-based drug-discovery techniques. Specifically, gefitinib, erlotinib, afatinib, and osimertinib are prominent examples of TKIs that benefited from structure-based drug-design approaches [1,17,20,21]. Furthermore, a significant portion of these TKI drugs were optimized from lead compounds using structure-based docking and analysis methods. For instance, afatinib and osimertinib underwent optimization through structure-based drug-design strategies, leveraging molecular docking and structure-activity relationship studies to enhance their potency and selectivity against EGFR mutations [17,21]. These examples underscore the pivotal role of structure-based drug discovery in both the development and optimization of TKI drugs targeting EGFR, contributing to their clinical efficacy and therapeutic success.

## 2. EGFR and Its Signal Transduction

**Figure 2:**
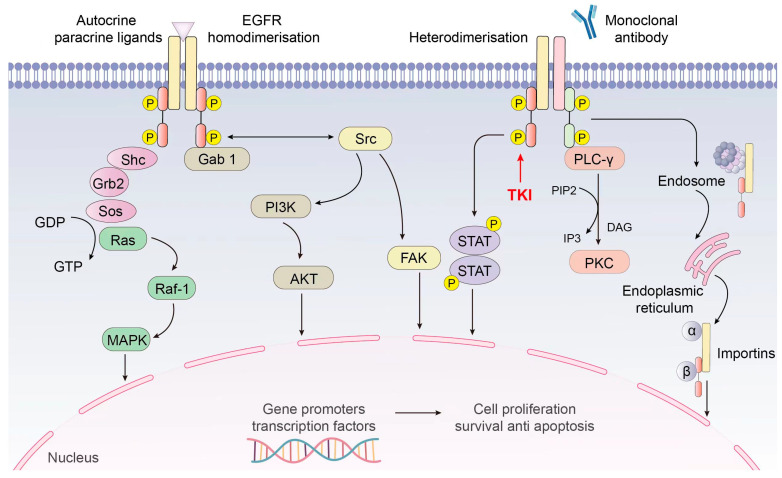
EGFR signal pathway.

EGFR signaling is a complex process involving a series of phosphorylation events and downstream signaling pathways. Here is a simplified overview of the signal transduction process—Ligand Binding (Figure 2): Upon ligand engagement, such as the union of EGF to the extracellular section of EGFR, a consequential conformational alteration in the receptor is initiated. Dimerization: The induced conformational alteration upon ligand binding to the extracellular domain of EGFR triggers and facilitates the dimerization process of the receptor, thereby effectively aligning the intracellular kinase domains in close spatial proximity. Auto-phosphorylation: The intracellular kinase modules inherent in EGFR catalyze the phosphorylation of distinct tyrosine residues present on the receptor itself. These phosphorylated tyrosine moieties function as pivotal sites for the recruitment and interaction with downstream signaling proteins [22]. Activation of downstream signaling: The activation of EGFR through phosphorylation of its tyrosine residues facilitates the recruitment of diverse adaptor proteins and enzymes. This assemblage notably encompasses pivotal components such as Grb2, Shc, and the consequential engagement of the Raf–Ras–MEK–MAPK pathway. Such interactions orchestrate intricate signaling cascades integral to the regulation of cellular growth and proliferation. Additionally, PI3K–Akt and STAT pathways are activated, influencing cell survival and gene expression [13]. Cellular responses: The varied cellular responses triggered by the activation of downstream signaling pathways following EGFR activation are indeed dependent on various factors, including the specific ligand, the context of cellular signaling, and the interplay between different signaling pathways. Ligand specificity: Different ligands binding to EGFR can activate distinct downstream signaling pathways. For example, while EGF primarily activates the MAPK pathway, other ligands like transforming growth factor alpha (TGF-α) may preferentially activate the PI3K–Akt pathway. This ligand specificity can lead to different cellular responses based on the particular ligand bound to EGFR. The cellular context, including the presence of other growth factors, cytokines, or environmental cues, can modulate the downstream signaling responses initiated by EGFR activation. For instance, the presence of certain growth factors may synergistically enhance or inhibit the effects of EGFR signaling, leading to context-dependent cellular responses. Interaction between pathways like MAPK and PI3K-Akt can modify cellular responses initiated by EGFR activation. Responses vary based on cell type and differentiation status, with EGFR signaling promoting proliferation in cancer cells but regulating normal cell growth and repair. In some cases, EGFR signaling can also influence cell migration and invasion [23].

## 3. Clinically Approved Representative Small-Molecule TKIs of EGFR

### 3.1. Sunvozertinib

Sunvozertinib, a novel TKI manufactured by Dizal Pharmaceuticals, represents an advancement arising from the need to overcome resistance mechanisms and thereby replaces previous generations of EGFR inhibitors. As marketed under the proprietary name DZD9008, this therapeutic entity embodies an innovative modality aimed at addressing NSCLC characterized by distinct mutations within the EGFR gene [24]. The pharmacological efficacy of sunvozertinib is predicated upon its specific capacity to inhibit EGFR with Exon 20 mutations, alongside targeting human epidermal growth factor receptor 2 (HER2) with Exon 20 insertions. This targeted inhibition is of significance due to the diminished responsiveness of cancer cells with these mutations to earlier generations of EGFR inhibitors. By competitively obstructing the ATP-binding site of these mutated tyrosine kinases, sunvozertinib effectively hinders the proliferative signaling pathways, exhibiting potent anti-tumor activity (ClinicalTrials.gov Identifier: NCT03974022). Preclinical models have demonstrated sunvozertinib’s capacity to inhibit tumor growth, especially in NSCLC models harboring Exon 20 insertions. Moreover, its substantiated ability to traverse the blood–brain barrier has provided favorable support for its prospective efficacy in managing central nervous system metastases. In clinical settings, sunvozertinib has shown promising efficacy, with ongoing trials further assessing its utility as a targeted intervention for individuals presenting specific EGFR mutations. The toxicity profile has been comparable to other EGFR inhibitors, with manageable side effects that do not significantly diminish its therapeutic value [25].

**Scheme 1:**
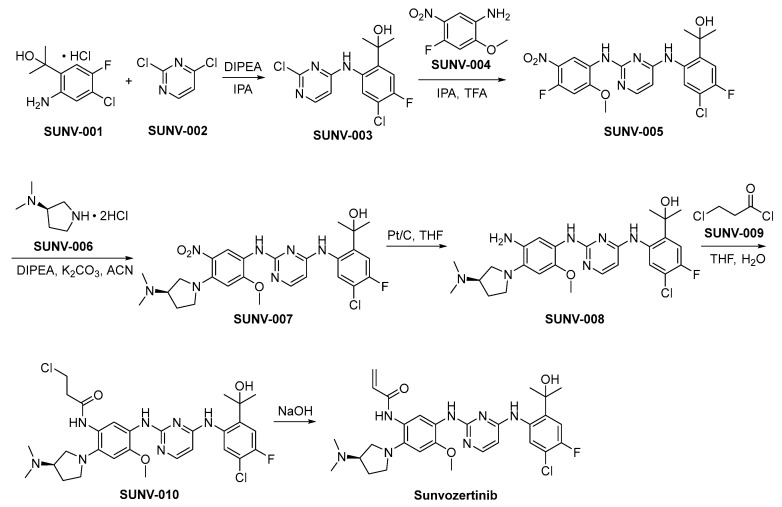
Synthesis of sunvozertinib.

The preparation of sunvozertinib is shown in Scheme 1 [26]. **SUNV-001** and **SUNV-002** engage in nucleophilic substitution reactions under alkaline conditions, yielding the formation of **SUNV-003** through a subsequent nucleophilic substitution process involving **SUNV-004**, ultimately leading to the generation of **SUNV-005**. **SUNV-005** and **SUNV-006** consecutively engage in nucleophilic substitution reactions, culminating in the formation of **SUNV-007**. The nitro moiety present in **SUNV-007** undergoes a reduction process to yield an amino functional group, through the utilization of hydrogen gas as the reducing agent and platinum carbon as the catalytic mediator, ultimately affording the formation of **SUNV-008**. The amino moiety present in **SUNV-008** and the acyl chloride functionality of **SUNV-009** engage in a condensation reaction, leading to the formation of the amide compound **SUNV-010**. **SUNV-010** is subjected to an elimination reaction in alkaline environments, leading to the formation of sunvozertinib.

### 3.2. Mobocertinib Succinate

Mobocertinib succinate, developed by Takeda Oncology, represents an orally administered TKI strategically engineered to selectively act upon EGFR with Exon 20 insertion mutations identified in NSCLC. Marketed under the brand name Exkivity, it was developed in response to the unmet medical need for effective treatments for NSCLC patients harboring these specific mutations [27]. Mobocertinib exhibits its therapeutic activity by selectively inhibiting the EGFR Exon 20 insertions, thereby obstructing aberrant signaling pathways that promote cancer cell proliferation. Its unique structure allows for targeted activity, minimizing the interaction with wild-type EGFR and reducing off-target effects [28]. Preclinical studies have shown that mobocertinib possessed significant antitumor efficacy in cell lines and animal models specifically engineered to express EGFR Exon 20 insertions, indicating its robust preclinical pharmacodynamics [29]. Clinically, mobocertinib has exhibited notable efficacy by eliciting tumor reduction in a distinct subgroup of individuals afflicted with advanced NSCLC featuring these infrequent genetic mutations. The endorsement by the U.S. Food and Drug Administration (FDA) stemmed from the outcomes observed in a comprehensive phase 1/2 clinical trial, emphasizing a noteworthy response rate and sustained response durability as pivotal determinants for approval [30]. Side effects noted include diarrhea, rash, and paronychia, which are consistent with the known toxicity profile of EGFR inhibitors.

**Scheme 2:**
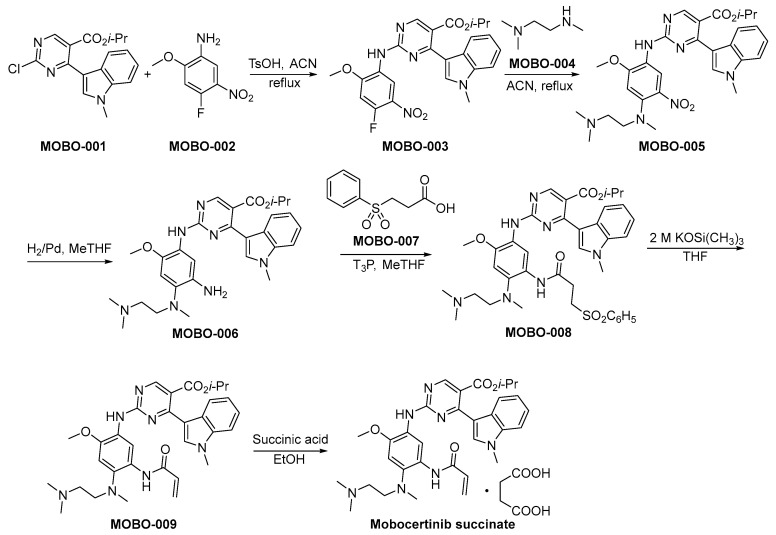
Synthesis of mobocertinib succinate.

The synthetic method of mobocertinib succinate is shown in Scheme 2 [31]. The compounds **MOBO-001** and **MOBO-002** are subjected to a nucleophilic substitution reaction, resulting in the formation of **MOBO-003**. Subsequently, **MOBO-003** engages in a nucleophilic substitution reaction with **MOBO-004**, yielding the synthesis of **MOBO-005**. The nitro moieties within **MOBO-005** undergo hydrogenation and subsequent reduction to amino functionalities employing palladium catalysis, yielding the compound denoted as **MOBO-006**. The condensation reaction between the amino group and carboxylic acid functional moieties, specifically denoted as **MOBO-007** and **MOBO-006,** respectively, culminates in the formation of **MOBO-008**. **MOBO-008** undergoes a process of elimination when exposed to robust alkaline conditions, resulting in the formation of **MOBO-009**. The compound **MOBO-009** was finally converted to the corresponding succinate.

### 3.3. Alflutinib

Alflutinib, also known as AST2818, represents a third-generation EGFR TKI strategically formulated for the therapeutic intervention in NSCLC. It is tailored to selectively address both the prevalent EGFR-sensitive mutations and the challenging T790M resistance mutation, acknowledged as pivotal pathogenic mechanisms in NSCLC progression [32]. Alflutinib was engineered to selectively bind to mutant forms of EGFR, sparing the wild-type receptor. This selectivity intends to optimize its anticancer efficacy by minimizing adverse effects attributed to the inhibition of wild-type EGFR in non-cancerous cells. In preclinical studies, alflutinib demonstrated potent activity against NSCLC cell lines harboring EGFR mutations, indicating promising preclinical pharmacodynamics. In diverse in vitro and in vivo models, the drug demonstrated efficacy, resulting in the attenuation of neoplastic proliferation. Clinical trials have reported that alflutinib exhibits substantial efficacy in individuals afflicted with NSCLC, particularly those carrying the EGFR T790M mutation, which is associated with resistance to earlier-generation EGFR inhibitors [33,34]. However, as with many TKIs, alflutinib has been associated with adverse events, including diarrhea and rash, though its toxicity profile is considered manageable with appropriate supportive care.

**Scheme 3:**
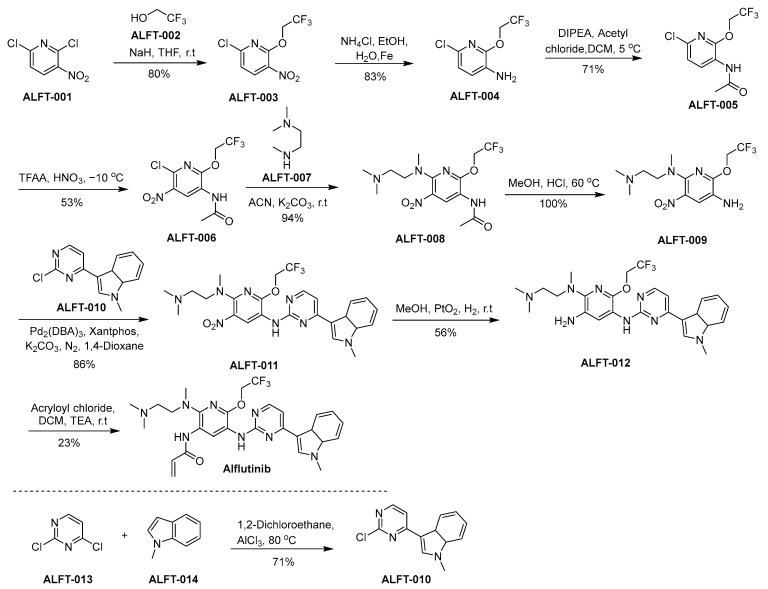
Synthesis of alflutinib.

The synthetic approach utilized for the synthesis of alflutinib is elucidated within the framework of Scheme 3 [35]. **ALFT-001** reacts with **ALFT-002**, in the presence of sodium hydride dissolved in tetrahydrofuran (THF), culminating in the formation of **ALFT-003**. Afterward, the nitro moiety within **ALFT-003** undergoes reduction, leading to the generation of **ALFT-004**. **ALFT-004** undergoes a chemical transformation upon reaction with acetyl chloride under the catalytic influence of N,N-diisopropylethylamine (DIPEA) in dichloromethane (DCM), affording the formation of **ALFT-005**. Subsequent nitration of **ALFT-005** using nitric acid and trifluoroacetic acid anhydride (TFAA) leads to the synthesis of **ALFT-006**. Subsequently, the compound **ALFT-006** engages in a chemical transformation with **ALFT-007**, yielding the product **ALFT-008**. A subsequent conversion of **ALFT-008** into **ALFT-009** is accomplished through treatment with methanol in the presence of hydrochloric acid (HCl). Next, the synthesis of **ALFT-011** is accomplished through the chemical transformation involving the reaction of **ALFT-009** with **ALFT-010**. The nitro moiety of Compound **ALFT-011** was reduced using hydrogen in the presence of platinum oxide as a catalyst to result in **ALFT-012**. In the final step of the synthetic pathway, **ALFT-012** undergoes a chemical transformation through its reaction with acryloyl chloride under the influence of triethylamine (TEA) within DCM, culminating in the emergence of alflutinib.

### 3.4. Lazertinib

Lazertinib, marketed as Leclaza, is a third-generation EGFR inhibitor specifically designed to target EGFR mutations, with a particular focus on the T790M mutation. It was developed to combat resistance mechanisms by selectively inhibiting both EGFR mutations and sparing wild-type EGFR to reduce off-target effects [36]. Mechanistically, lazertinib works by binding to the ATP-binding site of mutant EGFRs, preventing autophosphorylation and subsequent activation of downstream pro-survival signaling pathways. Preclinically, lazertinib showed a potent inhibition of tumor growth in cell lines and animal models possessing EGFR mutations, demonstrating effective pharmacodynamics. This drug demonstrated significant efficacy in clinical trials, particularly showing a positive response in NSCLC patients with EGFR mutations, specifically those who acquired T790M mutations after prior treatment with initial or second-generation EGFR inhibitors [37]. The toxicity profile of lazertinib has been noted to include manageable side effects typical of EGFR inhibitors, such as rash and diarrhea, without the severe cardiotoxicity or interstitial lung disease often associated with this drug class.

**Scheme 4:**
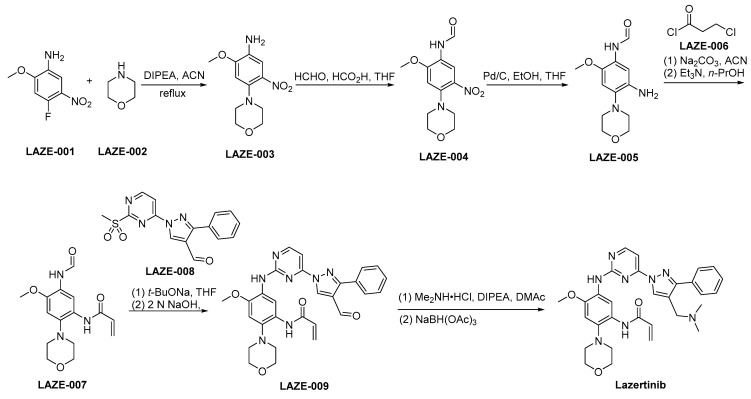
Synthesis of lazertinib.

The synthetic strategy employed for the preparation of lazertinib is outlined in Scheme 4 [38]. **LAZE-001** undergoes nucleophilic substitution by **LAZE-002**, leading to the formation of **LAZE-003**. Subsequently, the amino moiety within **LAZE-003** undergoes formaldehyde-mediated transformation, yielding **LAZE-004**. A reduction of the nitro group of **LAZE-004** via hydrogenation using Pd/C as a catalyst provides the corresponding aminoderivative **LAZE-005**. Following the condensation process, the compound **LAZE-005** and the acyl chloride **LAZE-006** experience elimination reactions when subjected to alkaline conditions, ultimately yielding the formation of **LAZE-007**. In the context of alkaline conditions, the compounds **LAZE-007** and **LAZE-008** engage in a condensation reaction, resulting in the generation of **LAZE-009**. Subsequently, the aldehyde moiety within the molecular structure of **LAZE-009** is subjected to reductive amination, resulting in the synthesis of lazertinib.

### 3.5. Dacomitinib

Dacomitinib, developed by Pfizer, represents a second-generation EGFR TKI. The mechanism of action for dacomitinib differs from that of first-generation TKIs as it forms an irreversible bond with the ATP binding site of the EGFR, resulting in a sustained inhibition of kinase activity. This irreversible binding mechanism is ascribed to the formation of a covalent bond with the cysteine residue situated within the ATP-binding pocket of EGFR, as well as its homologous family members HER2 and HER4 [39]. The FDA awarded dacomitinib (VIZIMPRO) with orphan drug designation in 2018. This endorsement specifically pertains to its primary application as a therapeutic option for individuals diagnosed with metastatic NSCLC featuring EGFR Exon 19 deletion or Exon 21 L858R substitution mutations [40]. Preclinical studies of dacomitinib showed potent activity against cancer cells harboring EGFR mutations and provided insight into its pharmacodynamics, indicating a longer duration of receptor downregulation compared to the first-generation TKIs [41]. Clinically, dacomitinib has exhibited efficacy in enhancing the duration of progression-free survival among individuals diagnosed with untreated NSCLC possessing EGFR mutations, surpassing the effects of gefitinib. In the ARCHER 1050 study, dacomitinib exhibited a notable extension in the median duration of progression-free survival [42]. However, dacomitinib’s toxicity profile includes diarrhea, dermatologic adverse effects, and mucosal inflammation, which are consistent with the inhibition of EGFR in normal tissues. These toxicities often require dose reductions and management with supportive care measures [43].

**Scheme 5:**
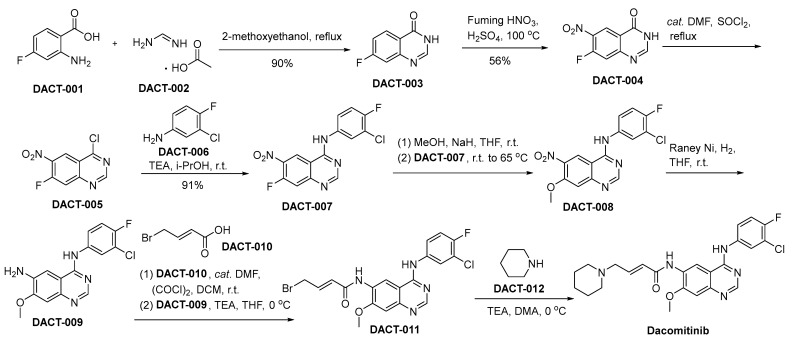
Synthesis of dacomitinib.

The synthetic pathway utilized for the synthesis of dacomitinib is depicted in Scheme 5 [44]. In the context of 2-methoxyethanol as the reaction medium, the chemical transformation involving 2-amino-4-fluoro-benzoic acid (referred to as **DACT-001**) with **DACT-002** culminates in the generation of a novel chemical entity denoted as **DACT-003**. **DACT-003** undergoes nitration using nitric acid to produce **DACT-004**. Subsequently, **DACT-004** is treated with thionyl chloride (SOCl_2_) in the presence of dimethylformamide (DMF) to produce **DACT-005** through chlorination. Following this, **DACT-005** engages in a substitution process with 3-chloro-4-fluoroaniline (**DACT-006**), facilitated by TEA within an isopropanol solvent milieu, ultimately yielding the compound designated as **DACT-007**. Upon subjecting **DACT-007** to methanol in the presence of sodium hydride, **DACT-008** is synthesized. A subsequent reduction of the nitro moiety within **DACT-008,** utilizing a Raney nickel catalyst, yields the **DACT-009**. Subsequent to this reduction, **DACT-009** undergoes a condensation reaction with **DACT-010**, leading to the synthesis of **DACT-011**. **DACT-011** undergoes a chemical reaction with piperidine (**DACT-012**), leading to the synthesis of the desired molecular entity, namely dacomitinib.

### 3.6. Pyrotinib Maleate

Pyrotinib maleate, sold under the brand name Hengrui, is a novel oral TKI designed to selectively target the EGFR and HER2. Developed by Jiangsu Hengrui Medicine Co., Ltd., it emerged from the need to treat HER2-positive breast cancers, which are often more aggressive and less responsive to hormonal therapy [45]. The drug acts by inhibiting the intracellular phosphorylation of HER2, leading to blockade of downstream MAPK and PI3K–Akt-signaling pathways, thereby impeding tumor cell growth and proliferation. Pyrotinib has displayed a broad range of inhibitory activity against HER2-overexpressing tumor cells in preclinical studies, with promising pharmacodynamic properties [46]. Clinically, pyrotinib has shown significant efficacy among individuals diagnosed with HER2-positive breast cancer. In a Phase II clinical investigation, it notably enhanced the objective response rate and median progression-free survival when contrasted against the combination of lapatinib and capecitabine in female patients grappling with advanced breast cancer [47]. The drug’s toxicity profile includes diarrhea, hand–foot syndrome, and hematologic toxicity. Despite these adverse effects, pyrotinib is generally well-tolerated when administered with appropriate supportive measures.

**Scheme 6:**
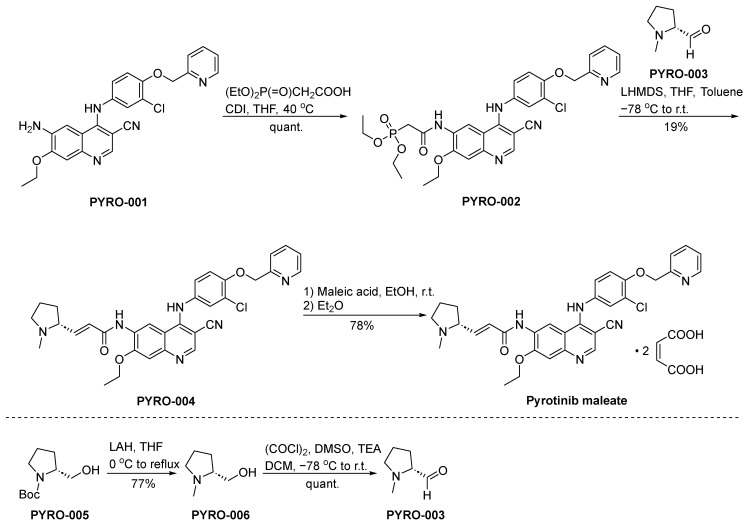
Synthesis of pyrotinib maleate.

The synthesis of pyrotinib maleate is depicted in Scheme 6 [48]. The condensation reaction of the aminoderivative **PYRO-001** and diethylphosphonoacetate leads to the formation of **PYRO-002**. Subsequently, **PYRO-002** and **PYRO-003** undergo a Wittig reaction, resulting in the synthesis of **PYRO-004**. The conversion of **PYRO-004** into pyrotinib maleate is achieved through the intervention of maleic acid.

### 3.7. Neratinib

Neratinib, branded as Nerlynx and developed by Puma Biotechnology, is an oral TKI targeting HER2 and EGFR. This drug was developed following the recognition of HER2’s pivotal involvement in the aggressive forms of breast cancer and the need for improved therapeutic options [49]. Neratinib irreversibly engages the ATP-binding cavity within these receptor proteins, resulting in the cessation of their kinase activity. Through the obstruction of signal transduction cascades, it interferes with cellular proliferation and viability, manifesting robust antineoplastic properties in preclinical model systems [50]. The drug showed clinical efficacy in the ExteNET study, a Phase III clinical trial where it significantly improved disease-free survival among early-stage patients afflicted with HER2-positive breast cancer who completed adjuvant trastuzumab therapy [51]. Toxicity profiles of neratinib include diarrhea, which is the most common adverse event, along with hepatotoxicity and rash. Effective management strategies, including a prophylactic use of anti-diarrheal medications, have been developed to mitigate these side effects.

**Scheme 7:**
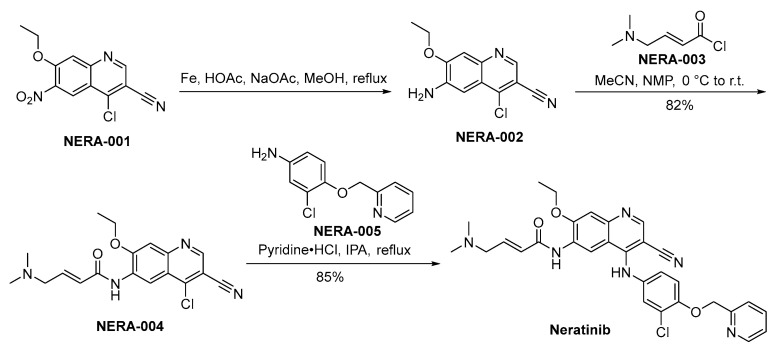
Synthesis of neratinib.

The synthetic methodology employed for the preparation of neratinib is disclosed in Scheme 7. The synthesis of neratinib is initiated through the reduction of **NERA-001** employing iron as the reducing agent, yielding the production of **NERA-002**. Following this, **NERA-002** undergoes a condensation reaction with **NERA-003**, resulting in the synthesis of **NERA-004**. The chloride moiety within the molecular structure of **NERA-004** undergoes displacement by **NERA-005**, culminating in the synthesis of neratinib [52].

### 3.8. Brigatinib

Brigatinib, commercially known as Alunbrig, was developed by Ariad Pharmaceuticals (now part of Takeda Oncology) and is a subsequent-generation inhibitor of anaplastic lymphoma kinase (ALK) [53]. The development of the compound was initiated in response to the emergence of resistance encountered with preceding ALK inhibitors within NSCLC [54]. Mechanistically, brigatinib exhibits selective binding affinity towards the ATP-binding domain of the ALK receptor tyrosine kinase, thereby inducing inhibition of ALK-mediated signaling pathways, consequently resulting in the demise of tumor cells. The drug is renowned for its ability to maintain efficacy against various ALK mutations associated with resistance to crizotinib, which belongs to the first generation of ALK inhibitors [55]. Preclinical studies demonstrated brigatinib’s potent activity against a range of ALK-positive tumor models, highlighting its potential pharmacodynamic benefits [56]. Clinically, discernible efficacy has been observed in individuals diagnosed with ALK-positive metastatic NSCLC, notably encompassing cases involving central nervous system (CNS) metastases [57]. The toxicity profile of brigatinib includes side effects such as elevated blood pressure, gastrointestinal disturbances, and the more severe, albeit less common, interstitial lung disease or pneumonitis. These adverse events necessitate careful patient monitoring [58].

**Scheme 8:**
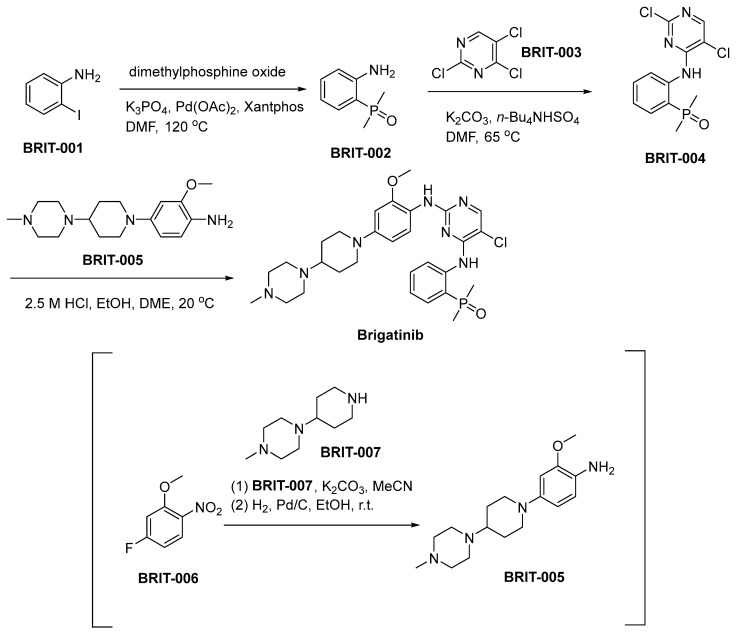
Synthesis of brigatinib.

The synthetic route utilized for the production of brigatinib is outlined in Scheme 8 [59]. The synthesis of **BRIT-002** entails the condensation of 2-iodoaniline (**BRIT-001**) with dimethylphosphine oxide, employing an adapted Hirao method featuring Xantphos as the ligand and palladium acetate as the catalyst. Subsequent to this stage, a nucleophilic aromatic substitution process occurs, characterized by the interaction between **BRIT-002** and trichloropyrimidine (**BRIT-003**), resulting in the synthesis of **BRIT-004**. The synthesis of aniline **BRIT-005** involves a nucleophilic aromatic substitution (S_N_Ar) process, wherein piperidine **BRIT-007** displaces the fluoronitroarene **BRIT-006**. This reaction pathway results in the formation of aniline **BRIT-005**. Subsequently, a catalyzed hydrogenative reduction of the nitro functional group is conducted, with Pd/C serving as the catalyst for this transformative chemical reaction. The ensuing transformation involves the reaction of **BRIT-004** with **BRIT-005**, culminating in the chemical synthesis of brigatinib.

### 3.9. Olmutinib

Olmutinib (brand name: Olita) was developed by Hanmi Pharmaceutical Co., Ltd. and later licensed to Boehringer Ingelheim for its potential in targeting EGFR mutations in NSCLC, particularly those patients with the T790M mutation, which induces resistance against initial-generation EGFR TKIs [60]. This mutation hinders the binding of earlier TKIs, prompting the need for development of third-generation inhibitors like olmutinib. The mechanism of action of Olmutinib involves irreversible binding to specific mutant EGFR, thereby inhibiting autophosphorylation and subsequent downstream signaling pathways. This ultimately leads to the inhibition of tumor cell proliferation and survival [61]. The efficacy of olmutinib against cell lines harboring the T790M mutation has been demonstrated in preclinical studies. The results of clinical trials demonstrated that olmutinib exhibited significant efficacy in patients diagnosed with T790M-positive NSCLC who experienced disease progression following prior TKI therapy, thereby providing a novel treatment option [62]. However, the clinical development of the drug faced challenges due to reports of serious adverse events such as severe skin toxicity and the rare but serious side effect known as Stevens–Johnson syndrome [63]. The development and distribution of the drug have been consequently limited, reflecting the paramount importance of safety in the risk–benefit assessment of novel cancer therapies.

**Scheme 9:**
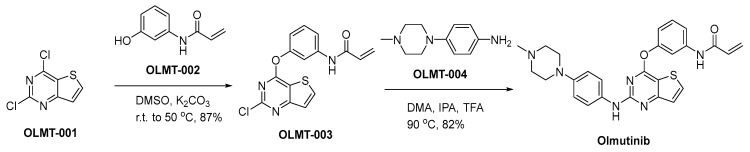
Synthesis of olmutinib.

A synthetic approach for the production of olmutinib is delineated within Scheme 9 [64]. The nucleophilic addition reaction of 2,4-dichloro-thieno[3,2-d]pyrimidine (**OLMT-001**) with N-(3-hydroxyphenyl)-2-propenamide (**OLMT-002**) is conducted employing potassium carbonate (K_2_CO_3_) as a base in dimethyl sulfoxide (DMSO), yielding the diaryl ether **OLMT-003**. In accordance with specialized reaction conditions characterized by an acid-mediated heating process, the process of introducing piperazinyl aniline **OLMT-004** into the synthesis pathway transpires. This reaction necessitates the concurrent presence of dimethyl adipate, isopropanol, and trifluoroacetic acid. This precisely orchestrated sequence culminates in the successful synthesis of the principal compound, known as olmutinib.

### 3.10. Osimertinib Mesylate

Osimertinib, commercially known as Tagrisso, is a meticulously engineered third-generation covalent EGFR inhibitor developed by AstraZeneca. It has been specifically designed to selectively target the prevalent EGFR T790M resistance mutation commonly encountered in NSCLC [65]. The development of osimertinib was undertaken as part of an endeavor to tackle the challenge posed by resistance observed in initial and subsequent generations of EGFR inhibitors. Osimertinib functions through selective inhibition, specifically targeting EGFR-TKI-sensitizing and EGFR T790M-resistance mutations. Its impact on wild-type EGFR demonstrates reduced activity, contributing to a discernibly diminished toxicity profile [17]. The preclinical studies demonstrated potent and selective pharmacodynamic activity against mutant EGFR forms, including the T790M mutation, in tumor models [66]. Osimertinib has exhibited a notable response rate in clinical settings for individuals suffering from NSCLC who have developed resistance to previous EGFR TKIs due to the T790M mutation. Additionally, it has demonstrated remarkable efficacy as a first-line treatment, significantly enhancing progression-free survival [67]. The safety profile of osimertinib is considered favorable, but it includes some risks such as cardiotoxicity, interstitial lung disease, and dermal side effects, although these are generally manageable [68].

**Scheme 10:**
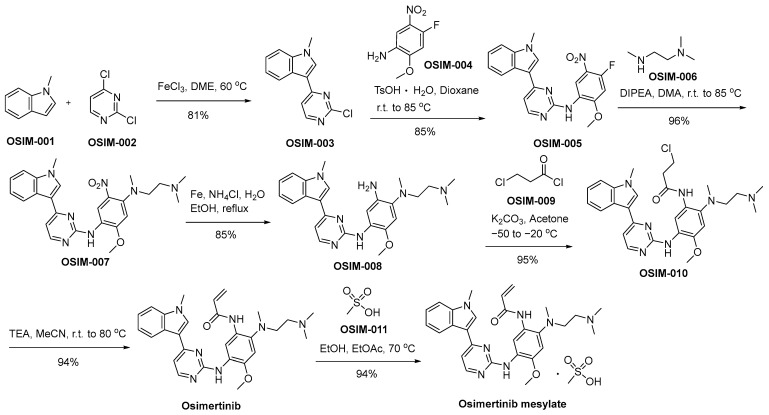
Synthesis of osimertinib mesylate.

Scheme 10 delineates a demonstrative synthetic method employed in the production of osimertinib mesylate, offering an illustrative framework for its synthesis [69]. The Friedel–Crafts arylation reaction involving N-methylindole (referred to as **OSIM-001**) and dichloropyrimidine (designated as **OSIM-002**) results in the formation of 3-pyrazinyl indole, denoted as **OSIM-003**. Subsequently, an S_N_Ar reaction with nitroaniline (identified as **OSIM-004**) is executed, affording the final product, aminopyrazine **OSIM-005**. The consecutive S_N_Ar reaction involving **OSIM-005** and N,N,N′-trimethylated ethylenediamine (**OSIN-006**) results in the formation of **OSIM-007**. Subsequently, **OSIM-007** undergoes a nitro reduction process facilitated by iron in an acidic milieu, ultimately yielding the triaminated arene compound recognized as **OSIM-008**. **OSIM-008** undergoes transformation into osimertinib through acylation utilizing 3-chloropropanoyl chloride (**OSIM-009**), followed by elimination in the presence of TEA within an acetonitrile milieu. Ultimately, the transformation of osimertinib is executed through a methanesulfonic acid-mediated treatment, denoted as **OSIM-011**, yielding the resultant product identified as osimertinib mesylate.

### 3.11. Afatinib Dimaleate

Afatinib, marketed as Gilotrif, is a second-generation TKI, developed by Boehringer Ingelheim, primarily for the treatment of NSCLC. Afatinib irreversibly inhibits the tyrosine kinases within the ErbB family, encompassing EGFR (ErbB1), HER2 (ErbB2), and ErbB4, and it also inhibits transphosphorylation of ErbB3 [21]. The objective behind the development of afatinib was to enhance outcomes for patients diagnosed with NSCLC with EGFR mutations. The inhibitory efficacy of afatinib was observed in preclinical investigations, effectively impeding signaling pathways arising from both homodimers and heterodimers across the ErbB family constellation. The observed phenomenon ultimately resulted in a clear inhibitory effect on the proliferation of tumor cells [5]. In clinical settings, afatinib has demonstrated efficacy as a viable first-line intervention for NSCLC patients harboring EGFR mutations, leading to improved progression-free survival compared to standard chemotherapy [70]. However, it is associated with side effects such as diarrhea, rash, and paronychia. Despite these toxicities, afatinib has been approved for use in several countries and is generally well tolerated with appropriate supportive care [71].

**Scheme 11:**
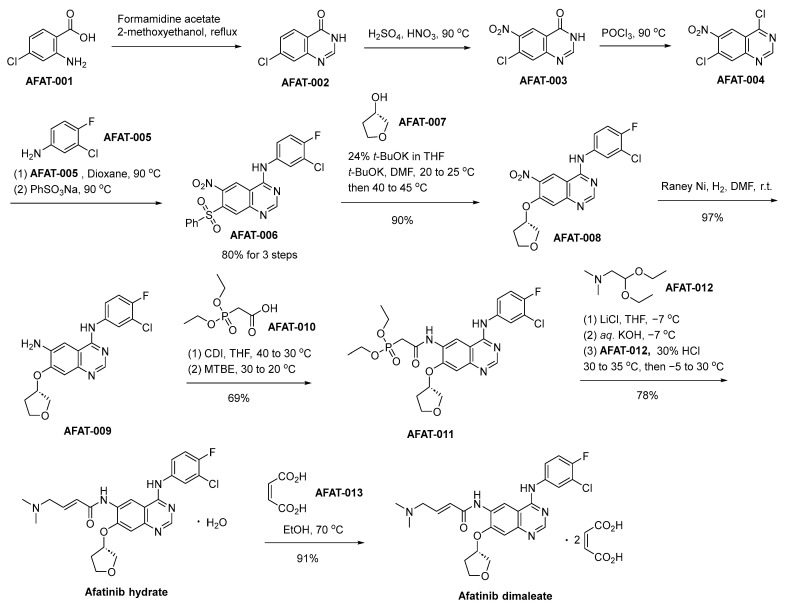
Synthesis of afatinib dimaleate.

The synthetic process for the production of afatinib dimaleate commences with the initial reaction involving 2-amino-4-chlorobenzoic acid (designated as **AFAT-001**) and formamidine acetate, thereby affording the intermediate compound denominated as **AFAT-002** (Scheme 11) [72]. **AFAT-002** undergoes nitration, yielding the nitroquinazolinone **AFAT-003**. An ensuing chlorination reaction, employing phosphorus oxychloride (POCl_3_), is employed to effectuate the transformation of **AFAT-003** into the compound denominated as **AFAT-004**. **AFAT-006** is subsequently acquired through the chemical transformation of **AFAT-004** via a reaction involving the commercially accessible 3-chloro-4-fluoroaniline (**AFAT-005**), succeeded by sulfonylation using sodium benzenesulfonate. **AFAT-006** engages in a chemical reaction with (*S*)-tetrahydrofuran-3-ol, designated as **AFAT-007**, yielding the compound **AFAT-008**. Subsequently, a Raney–Nickel (Raney–Ni) catalyzed reduction of the nitro functional group ensues, leading to the generation of the aniline derivative denoted as **AFAT-009**. The treatment of **AFAT-009** with a combination of **AFAT-010** and N,N′-carbonyldiimidazole results in the generation of **AFAT-011**. Subsequently, employing the Horner–Wadsworth–Emmons homologation reaction scheme facilitates the transformation, ultimately affording the (*E*)-olefinic afatinib hydrate compound. Ultimately, the final step involves the treatment of afatinib hydrate with maleic acid, leading to the proficient synthesis of the desired compound, namely afatinib dimaleate.

### 3.12. Vandetanib

Vandetanib, commercially known as Caprelsa, is a TKI developed by AstraZeneca. Its FDA approval in 2011 specifically pertains to its application in the management of advanced medullary thyroid cancer (MTC). Vandetanib targets multiple tyrosine kinases, including EGFR, vascular endothelial growth factor receptor (VEGFR), and the rearranged during transfection (RET) proto-oncogene [73]. The principal mode of operation for vandetanib primarily revolves around the suppression of these kinases, which holds significant significance in the context of tumor angiogenesis and cellular proliferation. By blocking these pathways, vandetanib suppresses tumor growth and the formation of new blood vessels [74]. The preclinical pharmacodynamic studies demonstrated the potent anti-angiogenic and antitumor effects of vandetanib in various cancer models [75]. The clinical trials of vandetanib demonstrated promising outcomes in patients with advanced MTC, leading to its approval by the FDA. However, the use of this medication is associated with a range of adverse effects that necessitate careful monitoring and management, including diarrhea, rash, hypertension, and cardiac toxicity [76]. While vandetanib represents a significant advancement in the treatment of advanced MTC, its use is limited to this specific indication. It highlights the significance of targeted therapies and individualized medicine in oncology, where drugs like vandetanib can provide substantial clinical benefit in well-defined patient populations.

**Scheme 12:**
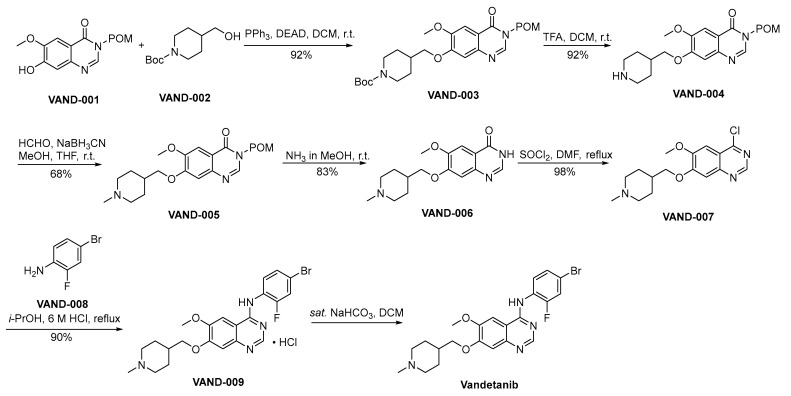
Synthesis of vandetanib.

Scheme 12 outlines the preparation methodology employed for the synthesis of vandetanib [77]. **VAND-001** and **VAND-002** engage in a Mitsunobu reaction, yielding **VAND-003** as the resultant product. Subsequently, **VAND-003** undergoes a deprotection process involving the removal of the Boc protective group from amino moieties, executed under acidic conditions, thereby affording the formation of **VAND-004**. The amino moiety residing within **VAND-004** is subjected to a reduction amination process catalyzed by formaldehyde, yielding the formation of **VAND-005**. **VAND-005** facilitates the deprotection of the POM protective group through a reaction conducted in the presence of methanolic ammonia. This process yields **VAND-006** as the resultant product. **VAND-006** undergoes a chlorination process catalyzed by thionyl chloride, resulting in the formation of **VAND-007**. **VAND-007** and **VAND-008** engage in a nucleophilic substitution reaction, resulting in the formation of **VAND-009**. Subsequently, **VAND-009** undergoes an alkaline treatment process, ultimately affording the synthesis of vandetanib.

### 3.13. Lapatinib Ditosylate

Lapatinib ditosylate, recognized by the brand Tykerb, represents a TKI developed by GlaxoSmithKline (GSK). In 2007, it obtained authorization from the FDA for managing advanced or metastatic breast malignancy [78]. The drug primarily targets HER2 and EGFR, both of which are overexpressed or mutated in certain types of breast cancer. The mechanism of action of lapatinib involves the inhibition of receptor phosphorylation, thereby obstructing downstream signaling pathways implicated in cellular proliferation and survival. The implementation of this targeted approach effectively regulates the proliferation of cancer cells [79]. The preclinical pharmacodynamic studies demonstrated that lapatinib exhibited potent inhibition of HER2 and EGFR signaling, resulting in a significant reduction in tumor growth in breast cancer models [80]. The efficacy of this treatment in managing HER2-positive metastatic breast cancer has been demonstrated through clinical trials, both as a standalone therapeutic regimen and when used in conjunction with adjunctive treatments. However, like many cancer drugs, lapatinib is associated with potential side effects, including diarrhea, skin rash, and cardiotoxicity, which require monitoring and management during treatment [81]. The development and approval of lapatinib represent a significant milestone in personalized medicine, as it specifically targets HER2-positive breast cancer. It underscores the importance of identifying and targeting specific molecular markers in cancer therapy, leading to more effective treatments for patients.

**Scheme 13:**
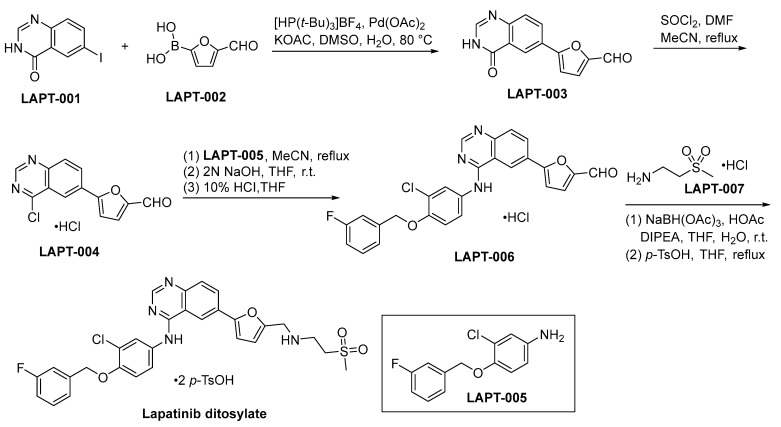
Synthesis of lapatinib ditosylate.

The initiation of lapatinib ditosylate synthesis is instigated through a Suzuki coupling reaction involving the precursors **LAPT-001** and **LAPT-002**. This catalytic event engenders the formation of an intermediate compound **LAPT-003**, as comprehensively explicated within Scheme 13. Subsequent to the preceding step, the chlorination of **LAPT-003** is conducted employing a reagent system composed of SOCl_2_ and DMF within a refluxing acetonitrile medium. This procedure yields the compound **LAPT-004** [82]. Subsequent to the initial step, the chlorinated functional group residing in **LAPT-004** is subjected to a substitution process with **LAPT-005**, resulting in the formation of **LAPT-006**. The ultimate stage in the synthetic route entails the reductive amination process of **LAPT-006** and **LAPT-007**, subsequently subjecting the resultant compound to treatment with para-toluenesulfonic acid (p-TsOH), leading to the conclusive formation of lapatinib ditosylate.

### 3.14. Erlotinib Hydrochloride

Erlotinib, marketed as Tarceva, is a TKI that has made a significant impact on cancer therapy. Developed by OSI Pharmaceuticals, it received FDA approval in 2004 for the management of NSCLC and subsequently expanded its therapeutic indications to include pancreatic cancer [83]. Erlotinib primarily targets the inhibition of EGFR, a pivotal cell surface receptor critically involved in regulating neoplastic cell proliferation and growth. Its mechanism of action involves inhibiting the activation of EGFR, thereby blocking downstream signaling pathways that are essential for cell division and survival. This targeted approach renders it highly effective against EGFR-mutated NSCLC and pancreatic cancer [3]. Preclinical pharmacodynamic studies have demonstrated the effective inhibition of EGFR signaling by erlotinib, resulting in reduced tumor growth in animal models. In clinical investigations, it has exhibited notable efficacy in improving overall survival and progression-free survival among individuals with advanced NSCLC and pancreatic malignancies, particularly those harboring distinct EGFR mutations [84]. Adverse effects associated with erlotinib include dermatological manifestations such as cutaneous rash, gastrointestinal disturbances, notably diarrhea, and fatigue, which can generally be managed with appropriate medical care. Severe adverse events like interstitial lung disease and liver toxicity are rare but necessitate close monitoring during treatment [85]. The development and clinical success of erlotinib underscores the significance of targeted therapies in oncology, where personalized treatments can offer substantial benefits for patients with specific genetic mutations. This has spurred the advancement of additional therapeutic modalities aimed at EGFR in the context of oncological interventions.

**Scheme 14:**
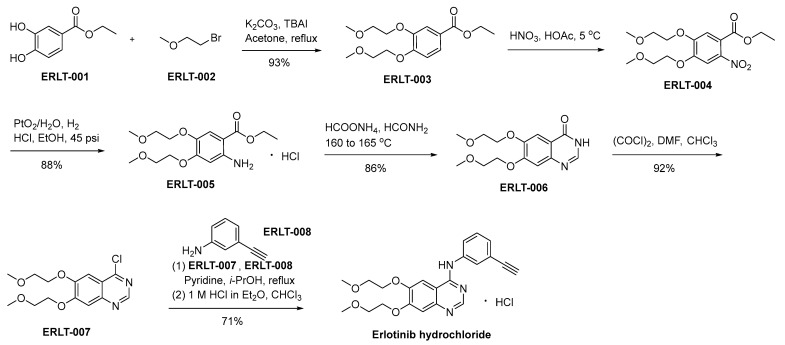
Synthesis of erlotinib hydrochloride.

In Scheme 14, a detailed delineation of the synthetic pathway for the production of erlotinib hydrochloride is meticulously expounded and presented [86]. **ERLT-001** undergoes a chemical transformation upon interaction with bromoethyl methyl ether (**ERLT-002**). In this synthetic procedure, potassium carbonate is used as the base, while tetrabutyl ammonium iodide (TBAI) functions as a catalytic agent. The result of this process yields **ERLT-003**, which subsequently undergoes a nitration step involving nitric acid, yielding the final product **ERLT-004**. The reduction of **ERLT-004** in an ethanol medium, catalyzed by PtO_2_/H_2_O under a hydrogen atmosphere, affords **ERLT-005**. **ERLT-005** experiences a cyclization process when subjected to specific reaction conditions comprising formamide and ammonium formate. This transformative reaction eventuates in the generation of the quinazolone derivative denominated as **ERLT-006**. Subsequently, the compound **ERLT-006** engages in a chemical transformation through its reaction with oxalyl chloride, yielding the **ERLT-007**. This intermediate undergoes a subsequent reaction with 3-ethynyl aniline, denoted as **ERLT-008**, facilitated by pyridine as a catalyst within an isopropanol environment. The resulting base was converted to the corresponding hydrochloride to result in erlotinib hydrochloride.

### 3.15. Gefitinib

The tyrosine kinase inhibitor Gefitinib, marketed as Iressa, has significantly impacted the therapeutic landscape of NSCLC. Originally developed by AstraZeneca, it received its first FDA approval in 2003 [87]. Gefitinib exerts its function through the inhibition of EGFR tyrosine kinase, thereby impeding downstream signaling pathways responsible for the proliferation of cancer cells [88]. Preclinical pharmacodynamic studies demonstrated the ability of gefitinib to effectively block EGFR signaling and inhibit tumor growth in animal models [20]. The efficacy of gefitinib has been further validated through clinical trials, particularly in individuals diagnosed with NSCLC carrying specific EGFR mutations. These mutations enhance the responsiveness of tumor cells to EGFR inhibitors [89]. Adverse events associated with gefitinib commonly manifest as dermatologic reactions, gastrointestinal disturbances such as diarrhea, and mild gastrointestinal discomfort. However, infrequent but critical incidences of interstitial lung disease and hepatic toxicity necessitate vigilant monitoring throughout the treatment regimen due to their severity. The development and clinical success of Gefitinib represent a significant advancement in targeted cancer therapy, showcasing the potential for personalized treatments based on genetic mutations. It has acted as a catalyst for further advancements in EGFR-focused therapeutic modalities within the field of oncology.

**Scheme 15:**
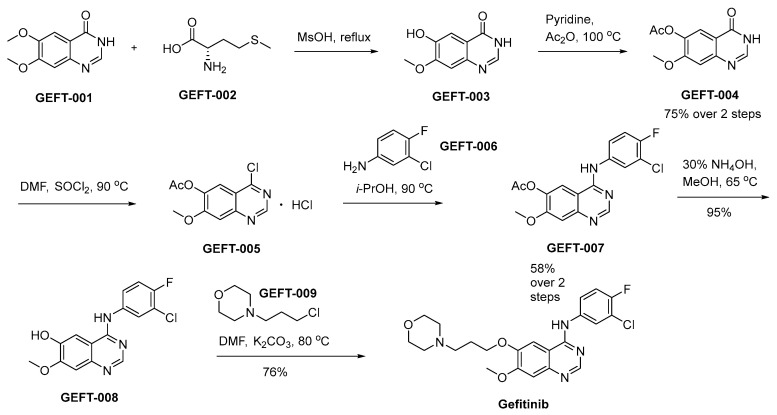
Synthesis of gefitinib.

The synthetic procedure initiates by the selective removal of the methyl group from the 6-methoxy group of **GEFT-001** by means of **GEFT-002** in methanesulphonic acid (Scheme 15) [90]. This orchestrated reaction pathway culminates in the generation of **GEFT-003**. Subsequent to the initial step, **GEFT-003** undergoes a chemical transformation involving acetic anhydride in the presence of pyridine, leading to the production of **GEFT-004**. Subsequently, this intermediate, **GEFT-004**, is subjected to a reaction with SOCl_2_ in the solvent DMF, ultimately affording the desired compound **GEFT-005**. The chlorine functional group present in **GEFT-005** undergoes substitution with **GEFT-006**, leading to the generation of **GEFT-007**. Subsequently, a hydrolytic transformation of **GEFT-007** into **GEFT-008** is achieved in the presence of ammonium hydroxide (NH_4_OH) under refluxing conditions in methanol. The interaction between **GEFT-008** and **GEFT-009**, facilitated by K_2_CO_3_ as the base in the solvent DMF, affords the successful synthesis of compound gefitinib.

### 3.16. Abivertinib

The novel TKI Abivertinib, marketed as Aociga, has been developed by Spectrum Pharmaceuticals and exhibits promising potential as a targeted therapy for various solid tumors, particularly NSCLC [91]. The primary target of the compound is located within EGFR and HER2. By inhibiting these receptor tyrosine kinases, abivertinib disrupts the intricate signaling cascades that facilitate cancer cell growth and proliferation [92]. Preclinical studies have demonstrated the potent inhibitory effects of abivertinib on EGFR and HER2 activation, leading to significant suppression of tumor proliferation in animal models. Ongoing clinical trials are currently underway to evaluate the compound’s safety profile and efficacy in oncology patients [93]. While abivertinib shows promise as a therapeutic option, similar to other targeted treatments, it may be associated with certain toxicities. Common adverse effects observed in clinical trials include skin rash, diarrhea, and fatigue. In line with other EGFR inhibitors, abivertinib potentially poses heightened concerns regarding severe adverse reactions such as interstitial lung disease and hepatotoxicity. The development and evaluation of abivertinib exemplify the ongoing efforts to discover and refine targeted therapies for cancer treatment. As research progresses and clinical trials continue, abivertinib holds potential to provide renewed hope for patients with EGFR- and HER2-driven cancers.

**Scheme 16:**
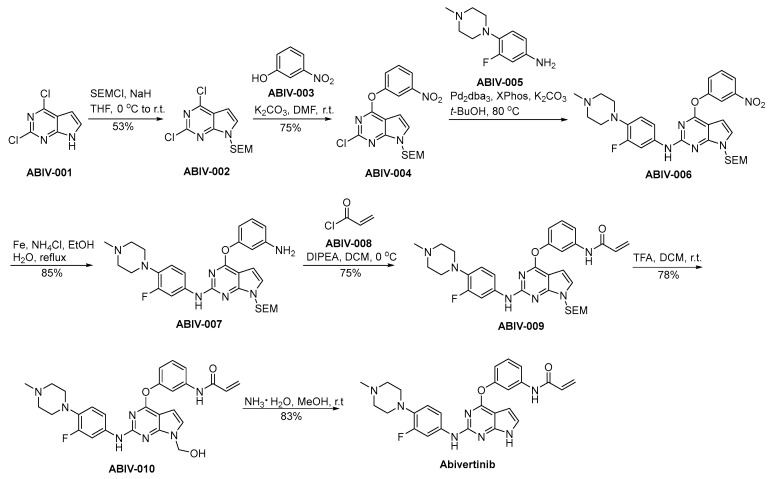
Synthesis of abivertinib.

The preparation procedure for abivertinib is presented in Scheme 16 [94]. **ABIV-001** is subjected to a safeguarding process employing SEM protective group under conditions of strong alkalinity, leading to the generation of **ABIV-002**. **ABIV-002** and **ABIV-003** partake in a nucleophilic substitution process, resulting in the generation of **ABIV-004**. Subsequently, **ABIV-004** experiences a nucleophilic substitution reaction with **ABIV-005**, affording the **ABIV-006**. **ABIV-006** undergoes reduction to form **ABIV-007**, which then reacts with acryloyl chloride **ABIV-008** through condensation to produce **ABIV-009**. The deprotection of the SEM safeguarding moiety in **ABIV-009** is achieved via acidic conditions, thereby affording the formation of hydroxyl functionalities, thereby yielding **ABIV-010**. **ABIV-010** undergoes a hydroxymethyl group elimination process mediated by ammonia, ultimately leading to the successful synthesis of abivertinib.

### 3.17. Poziotinib

Poziotinib is an orally administered TKI that has demonstrated significant potential in the therapeutic management of NSCLC. It is developed by Hanmi Pharmaceutical Co., Ltd. Specifically targeting EGFR mutations, including those associated with resistance to other EGFR inhibitors, Poziotinib offers a valuable therapeutic option [95]. The drug’s mechanism of action involves the inhibition of EGFR and other kinases responsible for signaling pathways pivotal to cellular proliferation and growth. What sets poziotinib apart from other EGFR inhibitors is its unique ability to selectively target a diverse range of EGFR mutations, including Exon 20 insertions [96]. Preclinical studies have shown that poziotinib exhibits potent activity against various EGFR mutations, resulting in tumor regression in lung cancer models. Clinical trials have shown its efficacy in NSCLC patients with EGFR mutations, particularly those patients with Exon 20 insertions [97]. Like many targeted therapies, poziotinib is associated with side effects, including skin rash, diarrhea, and hepatotoxicity, which require careful monitoring and management during treatment. However, its potential to address EGFR-mutant NSCLC represents a significant advancement in personalized medicine for lung cancer patients. In summary, the development of poziotinib marks a crucial milestone in the treatment of EGFR-mutant NSCLC, particularly those with Exon 20 insertions. Its ability to target a broader spectrum of EGFR mutations offers renewed hope to patients who have previously encountered limited therapeutic options.

**Scheme 17:**
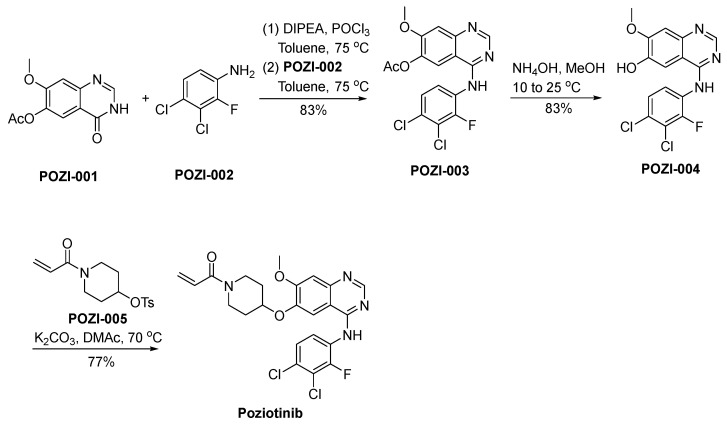
Synthesis of poziotinib.

The synthetic strategy employed for the preparation of poziotinib is outlined in Scheme 17 [98]. **POZI-001** undergoes a chlorination reaction, catalyzed by the reagent POCl_3_, subsequently proceeding through a nucleophilic substitution reaction with **POZI-002**, ultimately yielding the compound **POZI-003**. Following this, **POZI-003** experiences deacetylation in an alkaline environment, resulting in its transformation into **POZI-004**. The phenolic hydroxyl moiety residing within the molecular structure of **POZI-004** actively engages in a nucleophilic substitution reaction with **POZI-005**, ultimately culminating in the synthesis of poziotinib.

## 4. Challenges and Opportunities

The advent of EGFR inhibitors has revolutionized the therapeutic landscape for various cancers, particularly in the case of NSCLC. While these therapies offer substantial advantages, they also present distinctive challenges and opportunities. This discussion delves into the fundamental aspects of EGFR inhibitors in cancer treatment.

Challenges: Resistance Mechanisms: Despite initial responses, resistance to EGFR inhibitors inevitably develops. Acquired resistance mechanisms, such as secondary mutations (e.g., T790M) and bypass signaling pathways, limit the duration of response to first- and second-generation EGFR inhibitors [99]. Toxicities: skin rash, diarrhea, and hepatotoxicity are common side effects associated with EGFR inhibitors. These toxicities can impact patients’ quality of life and may necessitate dose adjustments or treatment interruptions [100]. Intratumoral Heterogeneity: Tumors often exhibit intratumoral heterogeneity, with different regions harboring distinct EGFR mutations. Targeting all subclones effectively remains a challenge [101]. CNS Metastases: EGFR-mutant NSCLC patients are prone to central nervous system (CNS) metastases. EGFR inhibitors have variable efficacy in addressing brain lesions owing to poor blood–brain barrier penetration [102].

Opportunities: Third-Generation EGFR Inhibitors: Third-generation EGFR inhibitors, exemplified by osimertinib, have shown efficacy against T790M resistance mutations. These agents extend progression-free survival and improve outcomes in NSCLC patients [67]. Combination Therapies: Combining EGFR inhibitors with other targeted agents or immunotherapies holds promise. Combinations like osimertinib plus savolitinib have demonstrated potential in addressing resistance mechanisms [103]. Next-Generation EGFR Inhibitors: Ongoing research is focused on developing next-generation EGFR inhibitors with enhanced efficacy and reduced toxicities. These agents aim to address existing challenges and improve patient outcomes. Liquid Biopsies: Liquid biopsies afford a non-invasive avenue for the surveillance of EGFR mutations and the identification of resistance mechanisms. An early detection of resistance can guide treatment decisions [19]. Patient Stratification: Advancements in molecular profiling enable precise patient stratification. Identifying specific EGFR mutations and resistance mechanisms helps tailor treatment strategies. Centralized CNS Therapy: Developing EGFR inhibitors with improved blood–brain barrier penetration can better manage CNS metastases, enhancing the overall effectiveness of these therapies [66]. EGFR inhibitors have transformed cancer treatment by targeting specific molecular alterations. While challenges like resistance and toxicities persist, ongoing research and the development of next-generation inhibitors and combination therapies provide opportunities to further improve patient outcomes. Patient stratification, liquid biopsies, and centralized CNS therapy are vital components of a comprehensive approach to addressing these challenges.

## 5. Conclusions

The synthesis and utilization of clinically approved small-molecule EGFR inhibitors in the field of cancer therapy represent a significant advancement towards precision medicine. These inhibitors have revolutionized oncology, providing targeted treatments for individuals affected by EGFR-driven malignancies, such as NSCLC and head and neck cancer. Despite substantial progress, persistent challenges remain in this field that require continued exploration and innovation. These challenges include the emergence of resistance mechanisms, potential off-target effects, synthetic complexity, patient stratification, and the need for effective combination therapies. Addressing these challenges necessitates rigorous scientific inquiry and clinical collaboration. Overcoming them requires the development of successive iterations of inhibitors with improved selectivity, better strategies to manage resistance, and enhanced patient-stratification techniques. The opportunities ahead are promising as personalized medicine gains momentum through advances in genomic profiling and non-invasive diagnostics enabling precise patient stratification. Next-generation EGFR inhibitors continue to evolve offering improved therapeutic options. Additionally, exploring combination therapies, including immunotherapies, holds immense potential for enhancing patient outcomes. As we navigate this dynamic landscape, it is essential for researchers, clinicians, and pharmaceutical scientists to work synergistically, leveraging their collective expertise to tackle challenges while maximizing opportunities. Regulatory entities play a crucial role in evaluating and authorizing these therapeutic interventions, prioritizing patient safety along with confirming efficacy. In summary, the synthesis and application of clinically approved small-molecule EGFR inhibitors signify a pivotal chapter in the ongoing battle against cancer. The journey is far from over, but the strides made in understanding EGFR-driven cancers and developing targeted therapies provide hope for improved patient outcomes and a future where precision medicine becomes the standard of care in oncology. Through continued dedication to research and innovation, we aim to better equip ourselves in the fight against this formidable disease and offer more effective treatment options to those in need.

## Figures and Tables

**Table 1:** Small-molecule EGFR inhibitors for cancer therapy ^a^.

NO	Drug	Company	Indications	Target	Status
1	Sunvozertinib	Dizal Pharmaceuticals (Shanghai, China)	NSCLC	EGFR; HER2;	Approved (2023)
2	Mobocertinib succinate	Takeda Oncology (Cambridge, MA, USA)	NSCLC	EGFR exon 20 insertion mutations;	Approved (2021)
3	Alflutinib	Shanghai Allist Pharmaceuticals Co., Ltd. (Shanghai, China)	NSCLC	EGFR T790M;	Approved (2021)
4	Lazertinib	Yuhan (Seoul, Republic of Korea) and Janssen Biotech (Titusville, NJ, USA)	NSCLC	EGFR; EGFR T790M; EGFR L858R; EGFR 19del;	Approved (2021)
5	Dacomitinib	Pfizer (New York, NY, USA)	NSCLC	HER2; HER4; EGFR; EGFR 19del; EGFR L858R;	Approved (2018)
6	Pyrotinib maleate	Jiangsu Hengrui Medicine Co., Ltd. (Lianyungang, China)	Breast cancer	HER2; HER4; EGFR;	Approved (2018)
7	Neratinib	Puma Biotechnology (Los Angeles, CA, USA)	Breast cancer	HER2; HER4; EGFR;	Approved (2017)
8	Brigatinib	Ariad Pharmaceuticals (San Diego, CA, USA)	NSCLC	EGFR; IGF1R; ALK; ROS; FLT3;	Approved (2017)
9	Olmutinib	Hanmi Pharmaceutical Co., Ltd. (Hwaseong-si, Gyeonggi-do, Republic of Korea)	NSCLC	EGFR T790M;	Approved (2016)
10	Osimertinib mesylate	AstraZeneca (London, UK)	NSCLC	EGFR; HER2; HER3; HER4; EGFR T790M; EGFR L858R; EGFR 19del;	Approved (2015)
11	Afatinib dimaleate	Boehringer Ingelheim (Dortmund, Germany)	NSCLC	HER4; HER2; EGFR; EGFR L858R; EGFR 19del;	Approved (2013)
12	Vandetanib	AstraZeneca (London, UK)	MTC	VEGFR2; RET; EGFR T790M;	Approved (2011)
13	Lapatinib ditosylate	GlaxoSmithKline (London, UK)	breast cancer	EGFR; HER2;	Approved (2007)
14	Erlotinib hydrochloride	Genentech (San Francisco, CA, USA)	NSCLC	EGFR; EGFR 19del; EGFR L858R;	Approved (2004)
15	Gefitinib	AstraZeneca (London, UK)	NSCLC	EGFR; EGFR 19del; EGFR L858R;	Approved (2002)
16	Abivertinib	Spectrum Pharmaceuticals (Irvine, CA, USA)	Acute respiratory distress syndrome	BTK; EGFR T790M;	Investigational
17	Poziotinib	Hanmi Pharmaceutical Co., Ltd. (Hwaseong-si, Gyeonggi-do, Republic of Korea)	NSCLC	HER2; HER4; EGFR;	Investigational

^a^ Company: Original company responsible for the development and marketing of the drug; indications: primary clinical indications; target: target of the drug’s action; status: the most recent updates regarding the research and development status of the drug, with the year provided indicating the approval date.

## Data Availability

Data are contained within the article.
